# Clinical value of the expression levels of tumor protein D52 and miR-133a on prognosis assessment of pancreatic cancer surgery

**DOI:** 10.12669/pjms.40.4.8389

**Published:** 2024

**Authors:** Zhen-yong Wang, Ru-hai Liu, Yu Meng, Jin-chao Li

**Affiliations:** 1 Zhen-yong Wang, The First Department of Hepatobiliary and Pancreatic Surgery, Cangzhou Central Hospital, Cangzhou 061000, Hebei, P.R. China; 2 Ru-hai Liu, The First Department of Hepatobiliary and Pancreatic Surgery, Cangzhou Central Hospital, Cangzhou 061000, Hebei, P.R. China; 3 Yu Meng, The First Department of Hepatobiliary and Pancreatic Surgery, Cangzhou Central Hospital, Cangzhou 061000, Hebei, P.R. China; 4 Jin-chao Li, The First Department of Hepatobiliary and Pancreatic Surgery, Cangzhou Central Hospital, Cangzhou 061000, Hebei, P.R. China

**Keywords:** Pancreatic cancer, Tumor protein D52, miR-133a, Prognosis

## Abstract

**Objective:**

To investigate the clinical value of the expression levels of tumor protein D52 (TPD52) and miR-133a on the prognosis assessment of pancreatic cancer surgery.

**Methods:**

This was a retrospective study. Ninety-seven patients who underwent radical surgery for pancreatic cancer in Cangzhou Central Hospital from January 2018 to January 2022 were selected and divided into four groups: TPD52 high expression group, TPD52 low expression group, miR-133a high expression group and miR-133a low expression group. The relationship between the expression levels of TPD52 and miR-133a and the clinicopathological features of patients with pancreatic cancer was analyzed. The COX regression model was used to analyze the risk factors affecting the prognosis of patients with pancreatic cancer.

**Results:**

The high expression rate of TPD52 and the low expression rate of miR-133a in pancreatic cancer tissues were higher than those in normal paracancerous tissues(P<0.05). Based on the comparison of prognosis and survival, the median survival time of patients with high expression of TPD52 and low expression of miR-133a was lower than that of patients with low expression of TPD52 and high expression of miR-133a, with a statistically significant difference(P<0.05). Moreover, multivariate Cox regression analysis showed that low differentiation of pancreatic cancer, III-IV stage of TNM, high expression of TPD52, as well as low expression of miR-133a were independent risk factors for postoperative survival of patients with pancreatic cancer(P<0.05).

**Conclusion:**

TPD52 is expressed at a high level whereas miR-133a at a low level in pancreatic cancer tissues, both of which together with low differentiation of pancreatic cancer and III-IV stage of TNM constitute independent risk factors affecting the surgical prognosis of patients with pancreatic cancer.

## INTRODUCTION

Pancreatic cancer, one of the deadliest digestive tract malignant tumors in the world, is experiencing a rapid increase in morbidity and mortality. Each year, pancreatic cancer claims the lives of about 200,000 people worldwide, and it ranks 6th and 7th among the causes of tumor-related deaths in men and women in China.^1^ Pancreatic cancer is clinically insidious, difficult to diagnose at an early stage, progresses rapidly with a very poor prognosis.^2^

Currently, surgical resection is the only possible cure for pancreatic cancer. However, its highly metastatic and aggressive nature causes most patients to be at an advanced stage at the time of diagnosis, so only a very small number of patients can be treated surgically. Studies have shown that the median survival time for those who did not undergo surgery was 3.5 months and the five years survival rate was only 6%, while both of them were only about 19 months and 25%, respectively, even after radical surgery.^3^,^4^

To this end, molecular marker evaluation for patients with pancreatic cancer is of positive effect on the treatment of pancreatic cancer. TPD52 (tumor protein D52) is a kind of proto-oncogene discovered in recent years, which has a close bearing on the occurrence, staging and infiltration degree of many tumors, with up-regulated expression in many tumors.^5^,^6^ MiRNA, as a kind of non-coding RNA^7^, is involved in many pathological and physiological processes such as cell growth, differentiation and carcinogenesis. In this study, the expression levels of TPD52 and miR-133a in tissue samples of patients with pancreatic cancer were detected to investigate the value of TPD 52 and Mir-133a in evaluating the prognosis of pancreatic cancer surgery.

## METHODS

This was a retrospective study. Paraffin-embedded tumor tissues and paracancerous tissues were selected from 97 patients who underwent radical surgery for pancreatic ductal adenocarcinoma in Cangzhou Central Hospital of Hebei Province from January 2018 to January 2022. All patients underwent radical surgery (68 cases of pancreaticoduodenectomy, 25 cases of distal pancreatectomy, four cases of total pancreatectomy, including eight cases of combined portal vein reconstruction).

### Ethical Approval

The study was approved by the Institutional Ethics Committee of Cangzhou Central Hospital [No.:2020-197-01(Z); date: December 09, 2020], and written informed consent was obtained from all participants.

### Inclusion criteria:

- Patients diagnosed as pancreatic ductal adenocarcinoma by postoperative pathology.
- Patients with complete perioperative data and valid data.

### Exclusion criteria:

- Patients who received other anti-tumor treatments including radiotherapy and chemotherapy before operation.
- Patients with other organ tumors or other malignant tumors of the pancreas.
- Patients with palliative resection.

### TPD52 detection method

Paraffin-embedded blocks were sliced and placed in a baking sheet at 65^0^C overnight. After dewaxing and ethanol hydration, they were soaked in phosphate buffer solution (PBS) for three times, five min each time. Citric acid antigen repair solution was used for antigen repair, and PBS was used for three times. Subsequently, it was incubated at room temperature of 3% H2O2 for 15 minutes, rinsed in PBS for three times, and added with goat serum dropwise, incubated at room temperature for 15 minutes, and rinsed in PBS for three times again. The first antibody (TPD52 rabbit anti-human polyclonal antibody, diluted at 1:100) was added dropwise, and it was kept in a wet box at 4^0^C overnight. On the next day, it was washed with PBS for three times, and the second antibody labeled with horseradish peroxidase (goat anti-rabbit IgG antibody, diluted at 1:500) was added, incubated at 37^0^C for 60 minutes and rinsed with PBS for three times. The color development of diaminobenzidine solution, hematoxylin double staining and neutral gum sealing were observed under the microscope.

### miR-133a detection method

The total RNA of tissues was extracted by TRIzol method, and RNA samples were qualified when the OD260/280 value of detected RNA was 1.8-2.0. According to the qRT-PCR reaction steps provided by the company, the two-step PCR amplification standard program was used for computer operation, and the relative expression level of miR-133a was calculated by 2^-ΔΔCT^ method. The experiment was repeated three times, and the average of the three experimental results was taken.

### Criteria for the expression of tpd52 and miR-133a:

TPD52 criterion: scored according to the percentage of positive cells, in which ≤5% was 0, 6%-25% was 1, 26%-50% was 2, and ≥51% was 3. According to the intensity of dyeing, score 0 means for no dyeing, one for light yellow, two for light brown, and three for dark brown particles. The staining intensity × the percentage of positive cells was the comprehensive score of immunohistochemistry, with 0-3 being low expression and 4-9 being high expression.

*Criteria for miR-133a:* The optimal cutoff value of miR-133a was determined by ROC curve, and pancreatic cancer patients were divided into miR-133a high expression group and miR-133a low expression group.

### Observation indicators

Clinicopathological features (gender, age, tumor site, tumor diameter, lymph node metastasis, liver metastasis, extrapancreatic nerve invasion, tissue differentiation, portal vein invasion, TNM staging), expression levels of TPD52 and miR-133a, and prognosis. Follow-up was conducted by telephone or outpatient service from the date of operation to the time of death or the last follow-up until August 31, 2022.

### Statistical analysis

All the data in this study were analyzed by SPSS21.0 software. The measurement data were expressed by mean ± standard deviation (*χ̅*;±*S*), and *t* test was used for comparison between groups. Counting data were expressed by the percentage of cases [n(%)], and the comparison between groups was made by χ^2^ test or Fisher exact probability method. GraphPad Prism 5 was employed to draw the survival curve, and Kaplan-Meier method and Log-rank test were used to compare the different trends of the two groups. Moreover, Cox risk regression model was used for multivariate survival analysis. P<0.05 indicates a statistically significant difference.

## RESULTS

The high expression rate of TPD52 and the low expression rate of miR-133a in pancreatic cancer tissues were higher than those in normal paracancerous tissues (P<0.05), Table-I.

**Table-I:** Comparison of expression levels of TPD52 and miR-133a innormal paracancerous tissues.

Group	TPD52		miR-133a	

	High expression	Low expression	High expression	Low expression
Pancreatic cancer tissue (n=97)	69(71.13)	28(28.87)	24(24.74)	73(75.26)
Normal paracancerous tissues (n=97)	30(30.93)	67(69.07)	90(92.78)	7(7.22)
*χ* ^2^	31.374		92.661	
*P*	0.000		0.000	

The expression level of TPD52 in pancreatic cancer had no obvious correlation with the patient’s gender, age, tumor location, tumor diameter, lymph node metastasis, liver metastasis and portal vein invasion (P>0.05), but was obviously related to whether the tumor has extrapancreatic nerve invasion, tumor differentiation degree and TNM stage (P<0.05). Besides, the expression level of miR-133a was not significantly correlated with gender, age, tumor location, tumor diameter, liver metastasis, extrapancreatic nerve invasion and portal vein invasion (P>0.05), but was significantly correlated with lymph node metastasis, tumor differentiation and TNM stage (P<0.05), Table-II.

**Table-II:** Expression of TPD52 and miR-133a in pancreatic cancer patients with different clinical features (n)

Clinical features	Cases	TPD52		χ^2^	P	miR-133a		χ^2^	P
	
		High expression	Low expression			High expression	Low expression		
Gender				3.478	0.062			1.290	0.256
Male	55	35	20			16	39		
Female	42	34	8			8	34		
Age (years old)				0.100	0.752			0.176	0.675
≥60	53	37	16			14	39		
<60	44	32	12			10	34		
Tumor site				1.289	0.256			0.953	0.329
Head and neck of pancreas	72	49	23			16	56		
Body and tail of pancreas	25	20	5			8	17		
Tumor diameter (cm)				0.038	0.846			0.031	0.861
≤4	50	36	14			12	38		
>4	47	33	14			12	35		
Lymph node metastasis				1.463	0.226			11.934	0.001
Yes	61	46	15			8	53		
No	36	23	13			16	20		
Hepatic metastases				0.030	0.862			1.372	0.242
Yes	4	3	1			0	4		
No	93	66	27			24	69		
Extrapancreatic nerve invasion				3.864	0.049			0.613	0.434
Yes	63	49	14			14	49		
No	34	20	14			10	24		
Degree of tissue differentiation				4.149	0.042			5.712	0.017
High-Middle	61	39	22			20	41		
Low	36	30	6			4	32		
Portal vein invasion				0.063	0.801			0.000	0.986
Yes	8	6	2			2	6		
No	89	63	26			22	67		
TNM staging				7.142	0.008			5.070	0.024
I-II	72	46	26			22	50		
III-IV	25	23	2			2	23		

**Fig.1:**
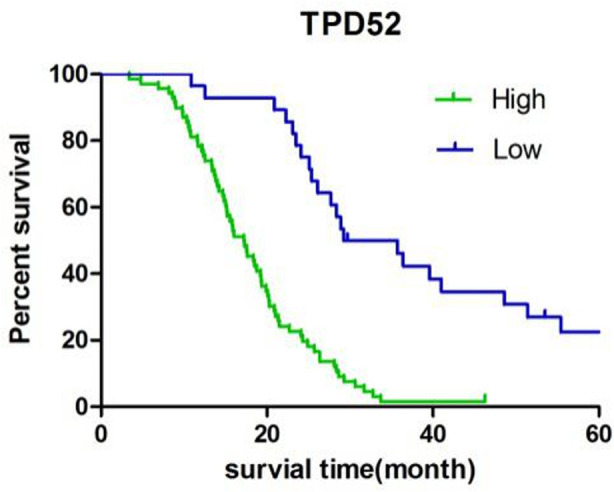
Kaplan-Meier survival curve of tpd52 expression.

**Fig.2:**
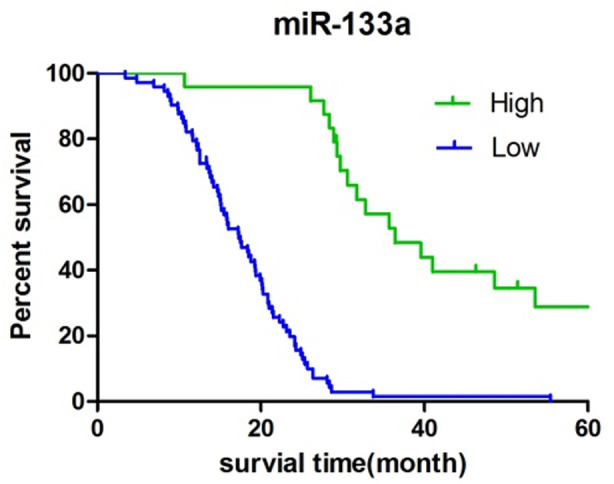
Kaplan-Meier survival curve of 2miR-133a expression

The 97 patients in this study survived 3.4-76.1 months from the time of surgery to the end of follow-up, with a median survival of 20.2 months. Up to the end of follow-up, seven cases were lost or no end-point events occurred. The median survival time of patients with high and low expression of TPD52 was 18.4 months and 34.7 months respectively, while that of patients with low and high expression of miR-133a was 17.2 months and 29.7 months respectively. The median survival of patients with high and low expression of TPD52 and miR-133a was compared, with a significantly significant different (P<0.05), Fig.1 and 2.

Kaplan-Merier survival analysis showed that lymph node metastasis, peripancreatic nerve invasion, tumor tissue differentiation, TNM staging and expression levels of TPD52 and miR-133a were all risk factors affecting the survival and prognosis of patients with pancreatic cancer (P<0.05), as shown in Table-III. Cox regression analysis showed that low differentiation of pancreatic cancer, III-IV stage of TNM, high expression of TPD52, as well as low expression of miR-133a were independent risk factors for postoperative survival of patients with pancreatic cancer (P<0.05), as shown in Table-IV.

**Table-III:** Univariate analysis of survival time of patients with pancreatic cancer after surgery (n).

Clinical features	Cases	Median survival time (months)	χ^2^	P
Gender			0.306	0.580
Male	55	21.0		
Female	42	18.8		
Age (years old)			1.445	0.229
≥60	53	21.5		
<60	44	18.7		
Tumor site			0.912	0.340
Head and neck of pancreas	72	19.9		
Body and tail of pancreas	25	20.9		
Tumor diameter (cm)			0.375	0.540
≥4	50	19.3		
<4	47	21.5		
Lymph node metastasis			19.198	0.000
Yes	61	18.4		
No	36	27.7		
Hepatic metastases			1.598	0.206
Yes	4	13.8		
No	93	20.3		
Extrapancreatic nerve invasion			4.934	0.026
Yes	63	18.5		
No	34	24.1		
Degree of tissue differentiation			26.309	0.000
High-Middle	61	24.9		
Low	36	15.5		
Portal vein invasion			0.033	0.986
Yes	8	20.1		
No	89	26.4		
TNM staging			37.338	0.000
I-II	72	23.5		
III-IV	25	16.5		
TPD52			33.340	0.000
High expression	69	17.2		
Low expression	28	29.7		
miR-133a			50.060	0.000
High expression	24	36.4		
Low expression	73	17.3		

**Table-IV:** Multivariate analysis of survival time of patients with pancreatic cancer after surgery (n).

Variable	Coefficient of regression	Standard error	Waldχ^ 2^	P	OR value	95%CI
Lymph node metastasis (Yes: No)	0.513	0.262	3.834	0.050	1.670	1.000-2.790
Extrapancreatic nerve invasion (Yes: No)	0.240	0.238	1.019	0.313	1.271	0.798-2.026
Degree of tissue differentiation (high-medium: low)	-0.991	0.256	15.013	0.000	0.371	0.225-0.613
TNM staging (I-II: III-IV)	-1.145	0.290	15.539	0.000	0.318	0.180-0.562
TPD52 (High: Low)	0.985	0.306	10.376	0.001	2.678	1.471-4.876
MiR-133a (High: Low)	-1.711	0.359	22.689	0.000	0.181	0.089-0.365

## DISCUSSION

It was shown in this study that the expression level of TPD52 in cancer tissues of patients with pancreatic cancer was higher than that in normal paracancerous tissues, and the expression level of TPD52 in patients with extrapancreatic nerve invasion, low differentiation of tumor and late TNM stage was higher than that in patients without extrapancreatic nerve invasion, high differentiation of tumor and early TNM stage(P<0.05), suggesting high expression level of TPD52 in patients with pancreatic cancer with high malignancy and late disease, which was consistent with some research results. It was shown in this study that the expression level of miR-133a in pancreatic cancer tissues was lower than that in normal paracancerous tissues (P<0.05), and the expression level of miR-133a in patients with lymph node metastasis, low differentiation and late TNM staging was lower than that in patients without lymph node metastasis, high-middle differentiation and high TNM staging(P<0.05), suggesting that low expression level of miR-133a in patients with high tumor malignancy and metastasis.

Although the treatment concept and surgical methods of pancreatic cancer are constantly updated, the prognosis of patients has not been significantly improved, and the status quo of diagnosis and treatment is still not optimistic.^8^,^9^ Traditional tumor markers lack specificity and sensitivity in the diagnosis of pancreatic cancer, and there is a lack of prognostic and curative effect evaluation indicators. It was pointed out in a study^10^ that the prognosis of patients with pancreatic cancer has a close bearing on tumor stage, pathological type and grade, whether metastasis occurs or not, etc. However, these clinicopathological indicators are still lack of specificity and can only be used to evaluate the condition in clinic, which fails to provide new choices and research directions for pancreatic cancer treatment targets. Therefore, finding new and more specific prognostic indicators and therapeutic targets for pancreatic cancer is beneficial to significantly change the current poor prognosis of pancreatic cancer.

TPD52, a member of tumor protein family D52^11^, was discovered in the research of breast cancer. It has four members, namely, TPD52, TPD52L1, TPD52L2 and TPD52L3, among which TPD52 is the most studied. Its gene is located in chromosome 8q21.13, which is the most common chromosome amplification region of tumor cells, and the encoded protein consists of about 200 amino acids. Studies have shown that the most common biological functions of TPD52 are regulating extracellular secretion and vesicle transport, participating in tumor proliferation, invasion and metastasis, inhibiting tumor cell apoptosis and DNA repair, and being highly expressed in various tumor tissues, such as breast cancer, ovarian cancer and prostate cancer.^12^-^14^ Currently, the role of TPD52 in breast cancer and prostate cancer is the most widely studied, and it has also been studied in other malignant tumors. The results show that TPD52 is closely related to the occurrence and development of tumors.^15^,^16^ However, it plays different roles in different tumors. For example, in renal cell carcinoma and primary hepatocellular carcinoma, TPD52 can inhibit the growth, development and metastasis of tumors, and is a tumor protective factor. In breast cancer, prostate cancer and acute myeloid leukemia, it promotes tumor invasion, and its expression level is negatively correlated with the survival period of patients.

MiRNA is a highly conserved non-coding single-stranded small molecule RNA, which participates in the occurrence, development, proliferation and metastasis of tumors by regulating gene expression. Its expression level has great influence on the biological behavior of tumors and has important diagnostic value. Its role in tumors has been widely studied.^17^,^18^ Studies have shown that miRNA affects the occurrence and development of pancreatic cancer, such as the proliferation ability of tumor cells, resistance to apoptosis, enhancement of angiogenesis, induction of metastasis and invasion.^19^ MiR-133a, as a member of miRNA, is also a key factor to regulate the cell activity of malignant tumor. At present, many studies show that it is beneficial to the body in most human tumors, and it is highly expressed in many tumors and has cancer inhibitory activity.^20^,^21^

Cox regression multivariate analysis showed that low differentiation of pancreatic cancer, TNM III-IV stage, high expression of TPD52 and low expression of miR-133a were independent risk factors affecting the prognosis of pancreatic cancer patients, which further confirmed that detecting the expression levels of TPD52 and miR-133a in pancreatic cancer tissues can predict the prognosis of pancreatic cancer patients and is expected to provide new targets for the treatment of pancreatic cancer.

### Limitations of the study

Nevertheless, this study was a single-center small sample study, fewer cases were included and it may affect the study results. To address this, a larger number of cases need to be included in later clinical work to further investigate the prognostic relationship between expression levels of TPD52 and miR-133a and patients with pancreatic cancer, the mechanism of TPD52 and miR-133a in the occurrence and development of pancreatic cancer also needs further study.

## CONCLUSIONS

TPD52 is expressed at a high level whereas miR-133a at a low level in pancreatic cancer tissues, both of which are related to the low differentiation, TNM III-IV stage and short survival time of patients with pancreatic cancer. Monitoring the expression levels of TPD52 and miR-133a in postoperative tissue samples is conducive to making a more accurate judgment on the prognosis of patients with pancreatic cancer.

### Authors’ Contributions:

**ZW** and **RL:** Carried out the studies, participated in collecting data, drafted the manuscript, are responsible and accountable for the accuracy and integrity of the work.

**YM: S**tatistical analysis and participated in its design.

**JL:** Acquisition, analysis, or interpretation of data and drafting the manuscript.

All authors read and approved the final manuscript.
